# Dual effects of a targeted small-molecule inhibitor (cabozantinib) on immune-mediated killing of tumor cells and immune tumor microenvironment permissiveness when combined with a cancer vaccine

**DOI:** 10.1186/s12967-014-0294-y

**Published:** 2014-11-13

**Authors:** Anna R Kwilas, Andressa Ardiani, Renee N Donahue, Dana T Aftab, James W Hodge

**Affiliations:** Laboratory of Tumor Immunology and Biology, Center for Cancer Research, National Cancer Institute, National Institutes of Health, 10 Center Drive; Room 8B13, Bethesda, MD 20892 USA; Exelixis, Inc., South San Francisco, CA USA

**Keywords:** Cabozantinib, Cancer vaccine, Immunotherapy, Combination therapy, Immune subset conditioning, Immunogenic modulation

## Abstract

**Background:**

Growing awareness of the complexity of carcinogenesis has made multimodal therapies for cancer increasingly compelling and relevant. In recent years, immunotherapy has gained acceptance as an active therapeutic approach to cancer treatment, even though cancer is widely considered an immunosuppressive disease. Combining immunotherapy with targeted agents that have immunomodulatory capabilities could significantly improve its efficacy.

**Methods:**

We evaluated the ability of cabozantinib, a receptor tyrosine kinase inhibitor, to modulate the immune system *in vivo* as well as alter the phenotype of tumor cells *in vitro* in order to determine if this inhibitor could act synergistically with a cancer vaccine.

**Results:**

Our studies indicated that cabozantinib altered the phenotype of MC38-CEA murine tumor cells, rendering them more sensitive to immune-mediated killing. Cabozantinib also altered the frequency of immune sub-populations in the periphery as well as in the tumor microenvironment, which generated a more permissive immune environment. When cabozantinib was combined with a poxviral-based cancer vaccine targeting a self-antigen, the combination significantly reduced the function of regulatory T cells and increased cytokine production from effector T cells in response to the antigen. These alterations to the immune landscape, along with direct modification of tumor cells, led to markedly improved antitumor efficacy.

**Conclusions:**

These studies support the clinical combination of cabozantinib with immunotherapy for the treatment of cancer.

**Electronic supplementary material:**

The online version of this article (doi:10.1186/s12967-014-0294-y) contains supplementary material, which is available to authorized users.

## Background

Cabozantinib, a small molecule inhibitor of multiple receptor tyrosine kinases (RTKs) [1], was approved in 2012 by the U.S. Food and Drug Administration (FDA) for the treatment of patients with progressive, metastatic medullary thyroid cancer [2]. Much of cabozantinib’s efficacy in this setting may derive from its inhibition of the RET RTK [3,4]. Cabozantinib also inhibits MET and VEGFR2, two RTKs believed to play major roles in the growth and dissemination of cancer cells. MET, the only known receptor for hepatocyte growth factor, functions to influence cell survival, proliferation, migration, and invasion. MET signaling can also affect tumor angiogenesis by stimulating VEGF and VEGFR expression, downregulating thrombospondin-1, and inducing tubulogenesis [5]. Mutation, amplification, and/or overexpression of the gene encoding MET has been observed in many tumor types, and in many cases has been associated with increased cancer aggressiveness, poor prognosis, and acquired resistance to standard therapies [6–11]. The VEGF pathway of tumor angiogenesis has been targeted extensively with antibody and small molecule inhibitor therapies [12–14]. However, in many cases, tumors overcome the initial inhibition of angiogenesis mediated by these therapies, in some cases due to an upregulation of MET signaling [15]. The dual inhibition of MET and VEGFR2 by cabozantinib may account for its activity in additional tumor types such as metastatic castration-resistant prostate cancer, renal cell carcinoma, and hepatocellular carcinoma, in which it is currently in phase III clinical trials [16–18].

Currently, the FDA has approved a limited number of immunotherapeutic agents for the treatment of cancer. These include sipuleucel-T, an autologous dendritic-cell vaccine for prostate cancer, ipilimumab, a monoclonal antibody that blocks the CTLA-4 inhibitory signal, and most recently pembrolizumab, a PD-1 inhibitor which also joins IL-2 and interferon-alpha for the treatment of melanoma [19–23]. However, many more immunotherapies, including poxviral-based cancer vaccines, are in late-stage clinical trials and are exhibiting substantial anti-tumor activity in multiple clinical settings [24–26].

Immunotherapeutic drugs have had positive clinical results as single agents. However, given the immunosuppressive nature of cancer, there is considerable room for improvement [27–29]. Combining immunotherapy with other targeted agents that have immunomodulatory capabilities in addition to antitumor properties has the potential to enhance its clinical benefit. Our studies have focused on two mechanisms for altering antitumor immune responses: immunogenic modulation and immune subset conditioning. Immunogenic modulation has been defined as the alteration of tumor cell phenotype such that the tumor cell becomes more sensitive to T cell-mediated lysis, most commonly through a modification of cell surface molecule expression. Standard therapies such as radiation, chemotherapy, and, most recently, androgen-deprivation therapy, have been shown to induce immunogenic modulation [30–33]. Immune subset conditioning involves altering the frequency and/or activity of immune cell subsets in the periphery and the tumor microenvironment, enabling more productive immune interactions leading to improved antitumor effects. Small molecule inhibitors have been shown to induce immune subset conditioning [34,35]. To date, however, no cancer therapy has been shown to mediate both immunogenic modulation and immune subset conditioning.

This study sought to investigate the ability of cabozantinib to improve the sensitivity of tumor cells to immune mediated lysis through immunogenic modulation and alter the immune landscape, both peripherally and in the tumor microenvironment (immune subset conditioning). We hypothesized that such effects could enable more productive immune interactions, potentially leading to synergistic antitumor effects when combining cabozantinib with a poxviral-based cancer vaccine. Here, we show that cabozantinib can (a) modulate the phenotype of tumor cells, making them more susceptible to T cell-mediated lysis, (b) modify the composition of the peripheral and tumor microenvironment immune compartments, (c) alter immune cell function, and (d) induce sustained CD4^+^ and CD8^+^ T cell-dependent tumor regression when combined with a poxviral-based cancer vaccine. Taken together, these findings suggest that cabozantinib is capable of both immunogenic modulation and immune subset conditioning, supporting its clinical combination with cancer immunotherapy.

## Results

### Cabozantinib reduced the proliferation of MC38-CEA tumor cells

**Figure 1:**
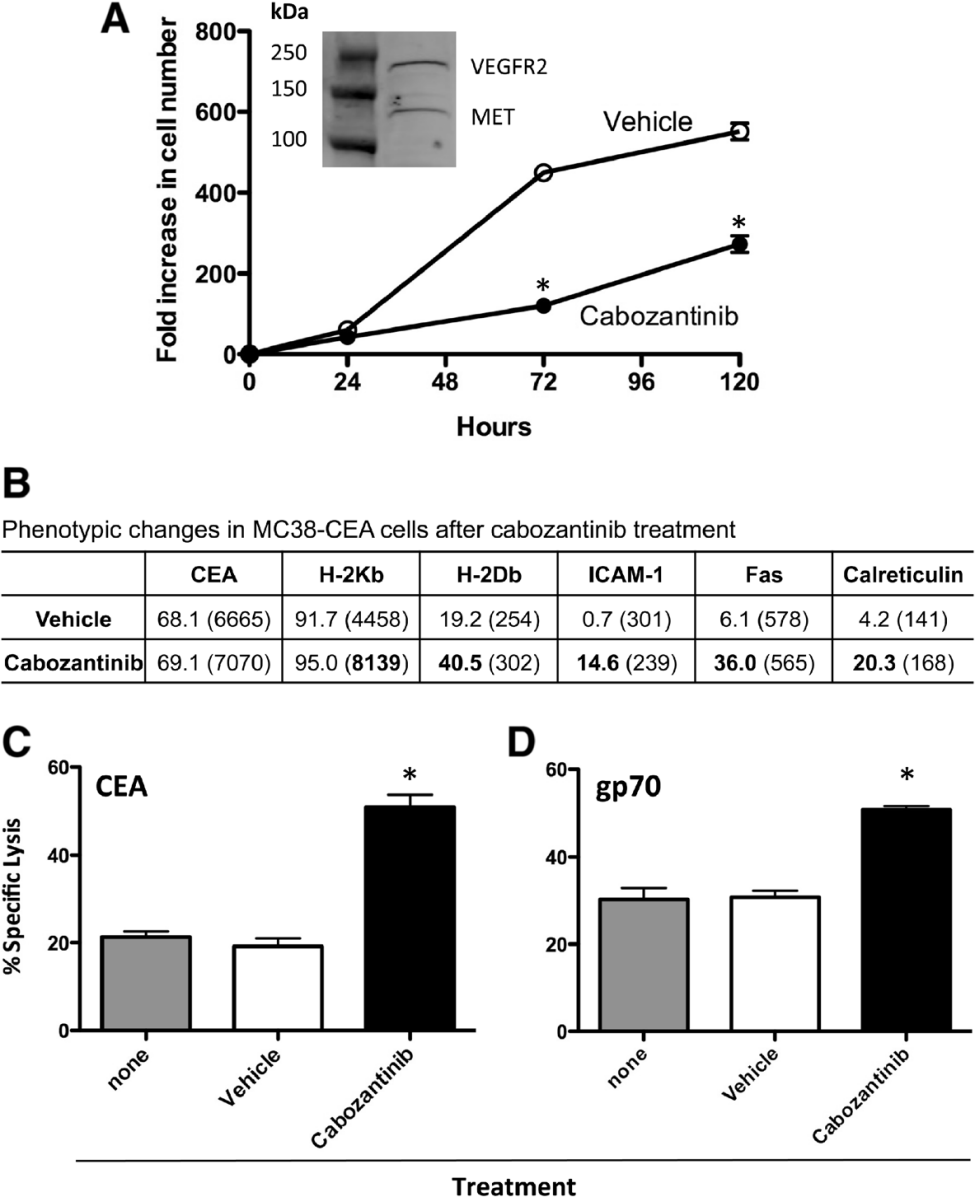
**Cabozantinib inhibits the growth, alters the phenotype and increases the sensitivity of MC38-CEA cells to T cell-mediated killing. (A)** MC38-CEA cells were treated with 2.5 μg/mL cabozantinib or vehicle for 1, 3, and 5 days then assayed for growth and viability. Inset panel: MET and VEGFR2 expression of MC38-CEA cells. **(B)** MC38-CEA cells were exposed to 2.5 μg/mL cabozantinib or vehicle for 24 h, then analyzed by flow cytometry for surface expression of CEA, MHC-I (H-2K^b^, H-2D^b^), ICAM-1, Fas, and calreticulin. Percent positivity and mean fluorescence intensity, in parentheses, are shown. Values in bold denote an increase of >40% relative to vehicle-treated cells. **(C, D)** MC38-CEA cells treated with cabozantinib (black bars) or vehicle (open bars) or left untreated (gray bars) for 24 h were labeled with ^111^In, then coincubated with CTLs specific for CEA **(C)** or gp70 **(D)** for 18 h at a ratio of 30:1. Bars indicate mean ± SEM. from quadruplicate measurements. Statistical analyses were done by Student’s *t* test. * = *P* <0.01. Data are representative of 3 independent experiments.

We used the MC38-CEA murine colon carcinoma cell line which expresses both VEGFR2 and MET (Figure 1A, inset), in addition to the human carcinoembryonic antigen (CEA), to examine the *in vitro* immunomodulatory effects and *in vivo* antitumor effects of cabozantinib. First, we determined the effect of cabozantinib on the proliferation of MC38-CEA cells. MC38-CEA cells were exposed to 2.5 μg/mL cabozantinib for 24, 72, or 120 h to model the steady-state plasma concentration achievable in humans [3]. After the designated period of treatment, cells were harvested and counted, and their viability was measured. Cabozantinib significantly reduced the proliferation of MC38-CEA cells after 72 and 120 h (Figure 1A). However, despite this reduction, the MC38-CEA cells continued to proliferate and their viability remained >75% at all time points, regardless of treatment. We therefore used this dose of cabozantinib for all subsequent *in vitro* studies.

### Cabozantinib modulated the expression of tumor cell markers associated with immune recognition

It has been previously shown that radiation and chemotherapy can alter the phenotype of tumor cells, rendering them more sensitive to T cell-mediated killing [31,32,36]. To determine if cabozantinib could modify the expression of cell-surface markers that influence immune recognition, we treated MC38-CEA cells with cabozantinib for 24 h, then stained and analyzed them by flow cytometry. Cabozantinib significantly upregulated the expression of MHC-I molecules on both the population level (H-2D^b^) and a per-cell basis (H-2K^b^), increasing the potential for antigen presentation and T cell recognition of the tumor cells (Figure 1B). Cabozantinib treatment also increased the percentage of MC38-CEA cells expressing ICAM-1, Fas, and calreticulin, cell-surface markers that also aid in T cell recognition, adhesion, and stimulation (Figure 1B). Prior studies have suggested that altering the expression of any one of these markers was capable of making tumor cells more amenable to T cell-mediated killing [31,32,36–38].

### Cabozantinib increased the sensitivity of MC38-CEA cells to T cell-mediated killing

To determine if cabozantinib treatment could increase the sensitivity of MC38-CEA cells to T cell-mediated lysis, we treated cells for 24 h, then used them as targets in CTL killing assays. Cabozantinib treatment significantly increased the sensitivity of MC38-CEA cells to T cell-mediated lysis by cytotoxic T lymphocytes (CTLs) specific for CEA (*P* =0.0004) (Figure 1C) as well as the endogenously expressed tumor antigen gp70 (*P* =0.0075) (Figure 1D). We next investigated whether cabozantinib treatment made tumor cells more susceptible to killing by other therapies or if their increased susceptibility was specific to T cell-mediated killing. We treated MC38-CEA cells with cabozantinib for 24 h, then exposed them to 0 or 5 Gy external-beam radiation and replated them in the absence of cabozantinib. Cells were counted and assayed for viability 24, 48, and 72 h post-irradiation. Administration of external-beam radiation further reduced the growth of cabozantinib-treated MC38-CEA cells, but did not reduce their viability as the cells remained >75% viable regardless of treatment (Additional file 1). Taken together, these data suggested that cabozantinib was capable of specifically altering tumor cells in ways that made them more amenable to immune mediated attack.

### Cabozantinib treatment altered the composition of the peripheral immune environment

**Figure 2:**
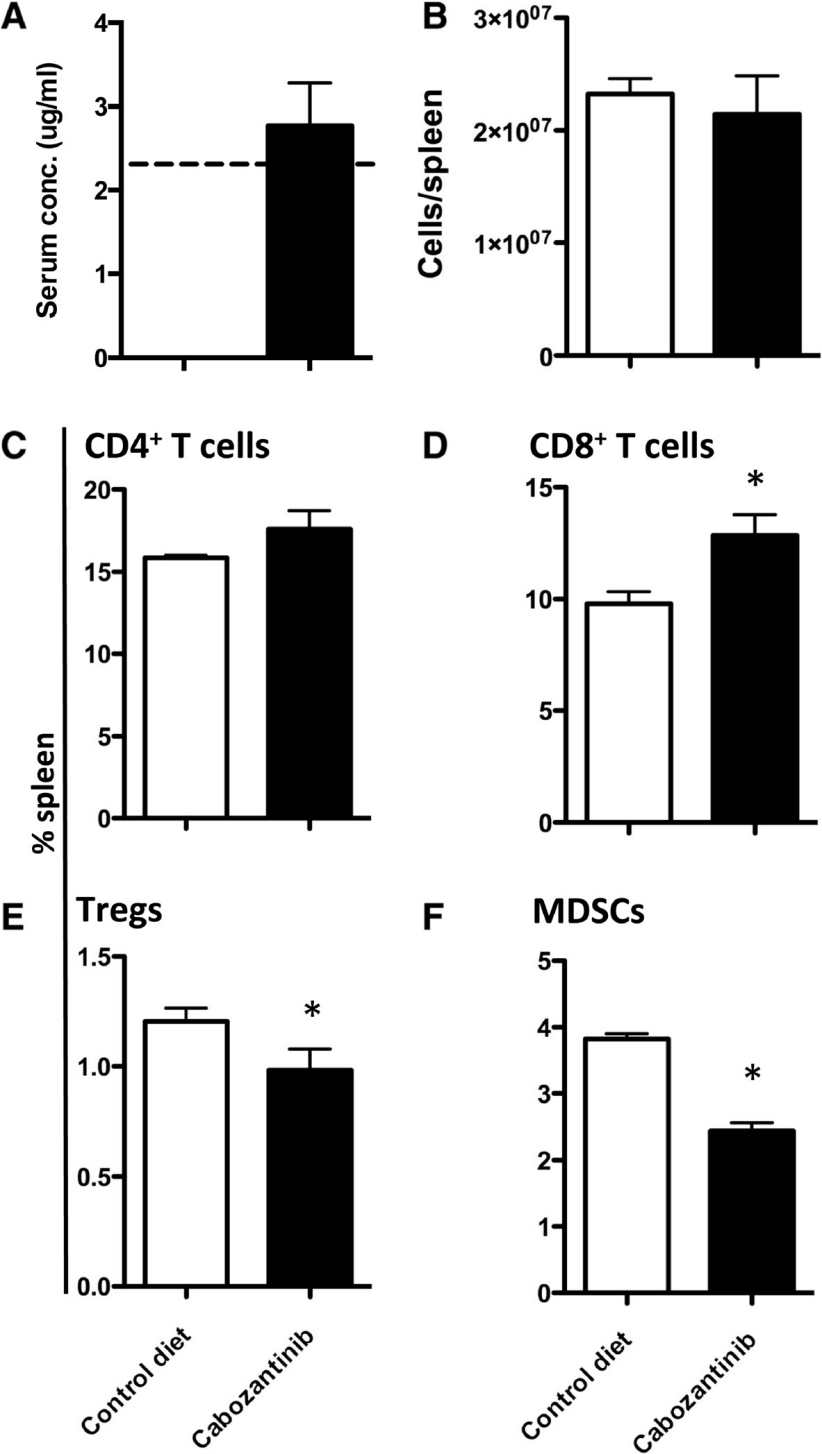
**Cabozantinib alters the immune-cell repertoire of C57BL/6 mice. (A)** Serum concentration of cabozantinib in mice fed control diet or diet containing cabozantinib for 10 days (*n* =5/group). Dashed line indicates the plasma concentration achieved at the maximum tolerated dose in a phase 1 human clinical trial. **(B)** Number of viable cells/spleen in mice fed control diet or cabozantinib diet for 35 days. The frequency of **(C)** CD4^+^ T cells (CD3^+^ CD4^+^), **(D)** CD8^+^ T cells (CD3^+^ CD8^+^), **(E)** Tregs (CD3^+^CD4^+^CD25^+^FoxP3^+^), and **(F)** MDSCs (CD11b^+^GR-1^+^) in spleens of mice fed control diet or cabozantinib diet for 35 days was determined by flow cytometry. Error bars indicate mean ± SEM. Statistical analyses were done by Student’s *t* test. * = *P* <0.05. Data are representative of 4 independent experiments.

To assess the impact of cabozantinib treatment on the peripheral immune environment, CEA-Tg C57/BL6 mice were fed a cabozantinib-compounded diet for up to 35 days. After 10 days on this diet, mice achieved a cabozantinib serum concentration of 2.77 ± 1.14 μg/mL, which was similar to the steady-state serum concentration achieved at the maximum tolerated dose in a phase I human clinical trial (2.31 μg/mL; Figure 2A) [3]. At 35 days, cabozantinib treatment did not affect the total number of splenocytes in treated mice (Figure 2B), nor did it significantly increase the percentage of CD4^+^ T cells in the spleen (*P* =0.1585) (Figure 2C). Cabozantinib treatment did, however, increase the percentage of CD8^+^ T cells (*P* =0.0198) (Figure 2D). In addition, treatment with cabozantinib reduced the percentage of T regulatory cells (Tregs) (*P* =0.0152) (Figure 2E), and myeloid-derived suppressor cells (MDSCs) (*P* <0.0001) in the spleen (Figure 2F). As a result of these alterations, CD4:Treg/MDSC ratios improved from 13.34 to 16.43 and 4.14 to 7.59, respectively even in the absence of a significant change in the level of CD4^+^ T cells. CD8:Treg/MDSC ratios improved from 8.24 to 11.91 and 2.54 to 5.50, respectively, due to the significant alteration of CD8^+^ T cells, Tregs, and MDSCs. These changes in immune-cell composition were evident as early as 10 days after initiating cabozantinib treatment. In addition, after 10 days of cabozantinib treatment we observed a transient increase in the percentage of CD4^+^ T cells (19.90% ±0.76 vs. 27.44% ±0.65; *P* <0.0001); however, this increase waned by day 35.

### Cabozantinib therapy synergized with a therapeutic cancer vaccine to improve T cell proliferation and function

**Figure 3:**
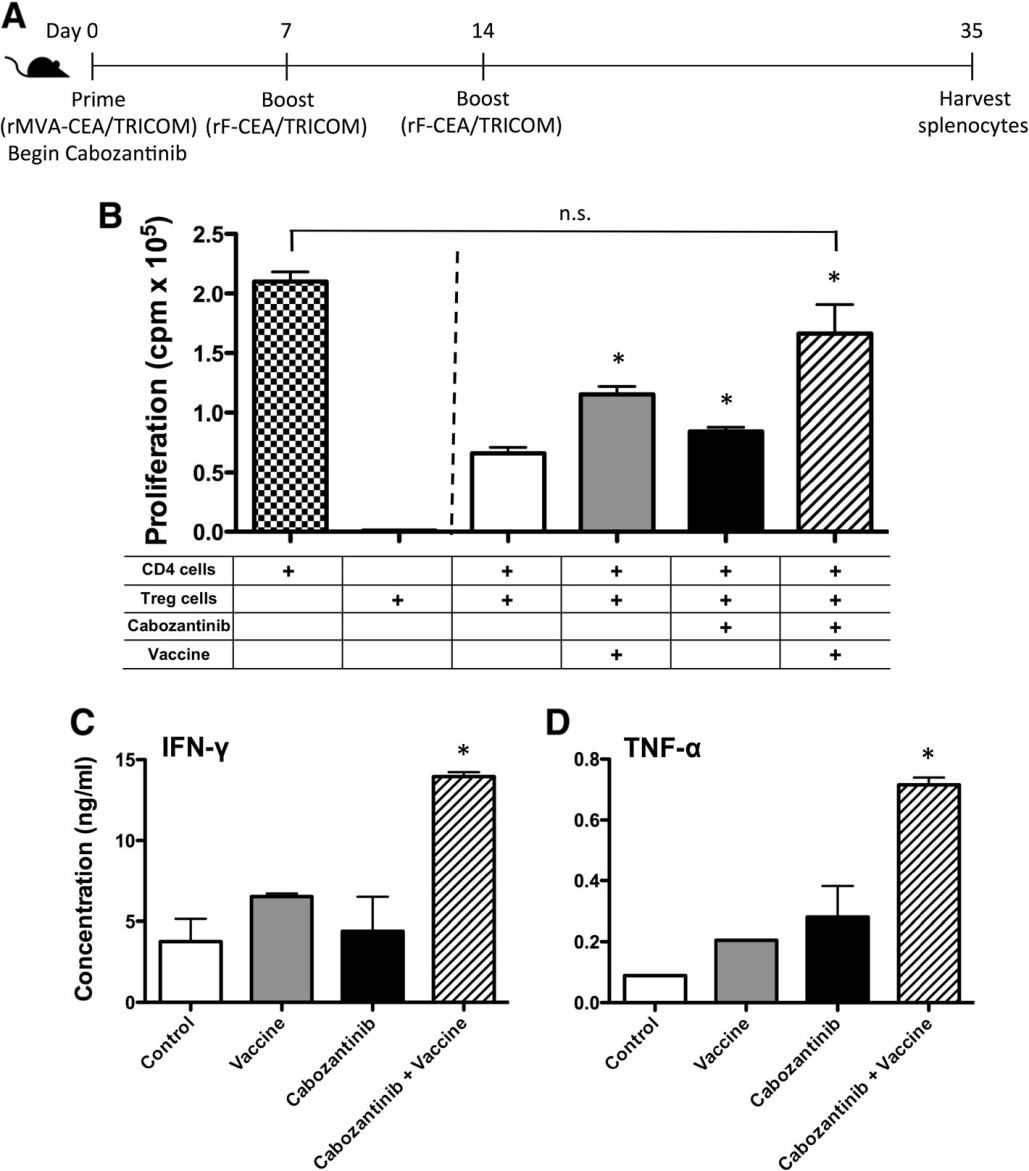
**Combining cabozantinib with a cancer vaccine improves antigen-specific immune responses in CEA-Tg C57BL/6 mice. (A)** Schema of combination therapy study (*n* =9). **(B)** To evaluate Treg function, Tregs (CD3^+^CD4^+^CD25^+^FoxP3^+^) were isolated from spleens and cocultured with CD4^+^ T cells from naïve mice, APCs (allogeneic splenocytes irradiated with 30 Gy), and soluble anti-CD3 for 72 h. Control wells containing Tregs, APCs, and anti-CD3 without CD4^+^ T cells were used to determine the background level of Treg proliferation. Control wells containing CD4^+^ T cells, APCs, and anti-CD3 without Tregs were used to determine the background level of CD4^+^ T-cell proliferation. Error bars indicate mean ± SEM. Statistical analyses were done by Student’s *t* test. * = *P* <0.01 relative to CD4^+^ T-cell proliferation in the presence of Tregs from untreated mice; n.s. indicates no significant difference between the proliferation of CD4^+^ T cells in the absence of Tregs or in the presence of Tregs from mice treated with combination therapy. (C, D) To evaluate CD8^+^ T-cell responses, harvested splenocytes were incubated with CEA peptide (1 μg/mL) for 7 days. Lymphocytes were then restimulated with fresh, irradiated, naïve splenocytes and 1 μg/mL of either CEA or HIV-gag peptide for 24 h. Supernatants were collected and analyzed for murine IFN-γ **(C)** and TNF-α **(D)** using a cytometric bead array. Nonspecific cytokine production in response to HIV-gag was subtracted from that induced by the CEA peptide. Error bars indicate mean ± SEM. Statistical analyses were done by Student’s *t* test. * = *P* <0.05 relative to control and single-agent treatments. Data are representative of 2 independent experiments.

After determining that cabozantinib could alter the peripheral immune landscape, we next combined it with a poxviral-based cancer vaccine to determine if the combination could alter the function of immune cells as well. Cabozantinib and MVA/rF-CEA/TRICOM were administered to CEA-Tg C57/BL6 mice as indicated in Figure 3A. After 35 days, splenocytes were harvested and Tregs were purified to assay their ability to regulate the proliferation of naïve CD4^+^ T cells. Compared to Tregs from control mice, Tregs from mice treated with cabozantinib alone demonstrated a significantly reduced ability to regulate the proliferation of CD4^+^ T cells (*P* =0.0183) (Figure 3B). Surprisingly, the regulatory capacity of Tregs from mice treated with MVA/rF-CEA/TRICOM alone was also significantly reduced (*P* =0.0003) (Figure 3B). However, the combination of cabozantinib and MVA/rF-CEA/TRICOM completely abrogated Treg function, as CD4^+^ T-cell proliferation in the presence of Tregs from these mice was not significantly different from that in the absence of Tregs (*P* =0.0775) (Figure 3B). Splenocytes from these mice were also analyzed for CEA-specific cytokine production. Splenocytes from mice treated with the combination of cabozantinib and MVA/rF-CEA/TRICOM produced significantly more interferon (IFN)-γ (Figure 3C; *P* =0.0188) and tumor necrosis factor (TNF)-α (Figure 3D; *P* =0.0015) than splenocytes from control mice. In addition, splenocytes from mice treated with the combination produced significantly more IFN-γ than splenocytes from mice receiving either cabozantinib alone (*P* =0.0463) or MVA/rF-CEA/TRICOM alone (*P* = 0.0020). Splenocytes from combination-treated mice also produced significantly more TNF-α than splenocytes from mice receiving MVA/rF-CEA/TRICOM alone (*P* =0.0022).

### Cabozantinib treatment reduced tumor vascularity and altered immune-cell infiltration when combined with a cancer vaccine

**Figure 4:**
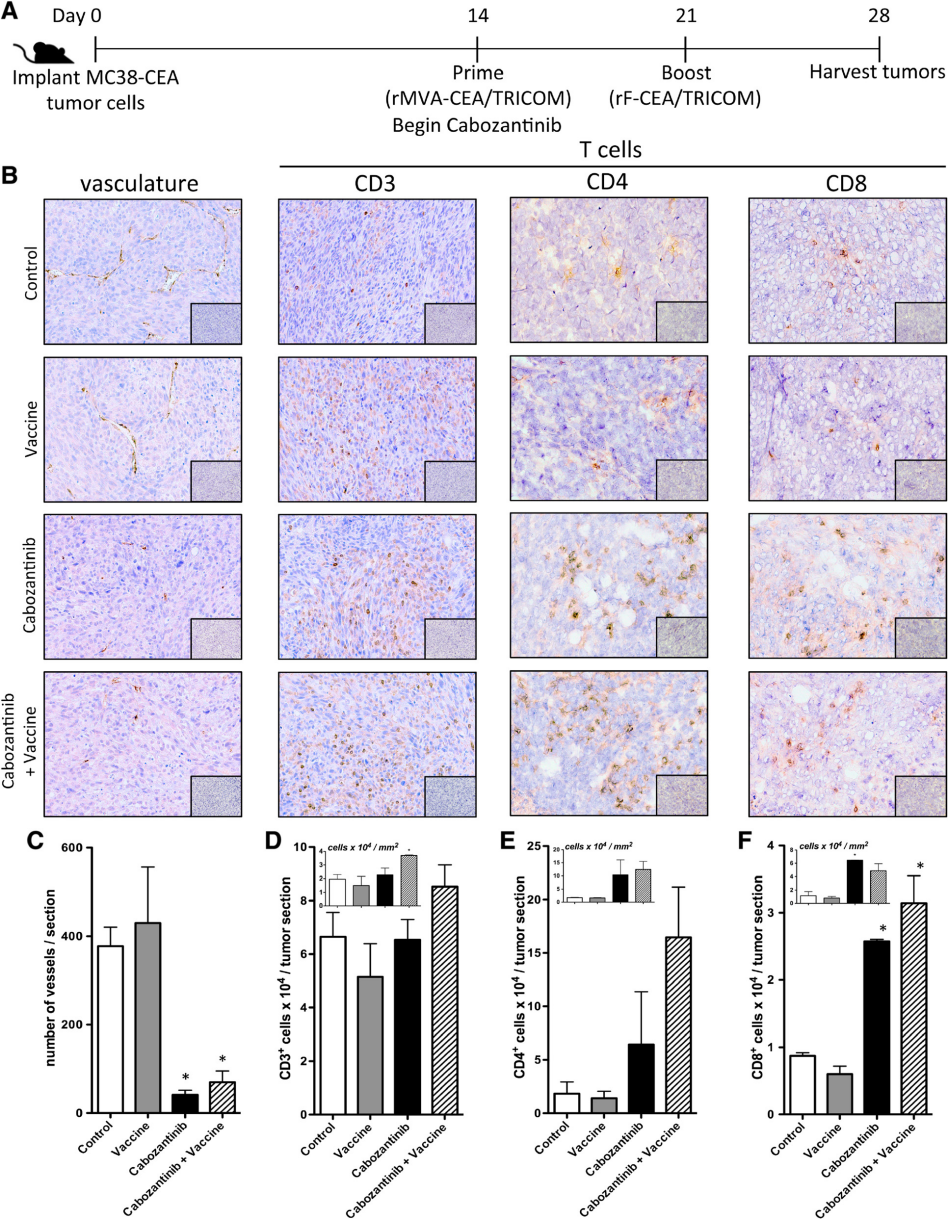
**Cabozantinib reduces tumor vascularity and improves T cell infiltration when combined with a cancer vaccine. (A)** Schema of combination therapy study (*n* =2/group). **(B)** Representative histological staining for indicated markers (top) on tumor sections from mice given indicated treatments (left). Inset panels: isotype staining. **(C–F)** Quantification of **(C)** blood vessels/tumor section; **(D)** number of CD3^+^ T-cells/tumor section; **(E)** number of CD4^+^ T-cells /tumor section; and **(F)** number of CD8^+^ T-cells /tumor section. Inset panels: number of indicated cells/mm^2^ of tumor. Marker staining was identified and enumerated using Aperio ImageScope image analysis software. Error bars indicate mean ± SEM. Statistical analyses were done by Student’s *t* test. * = *P* <0.05 relative to control.

**Figure 5:**
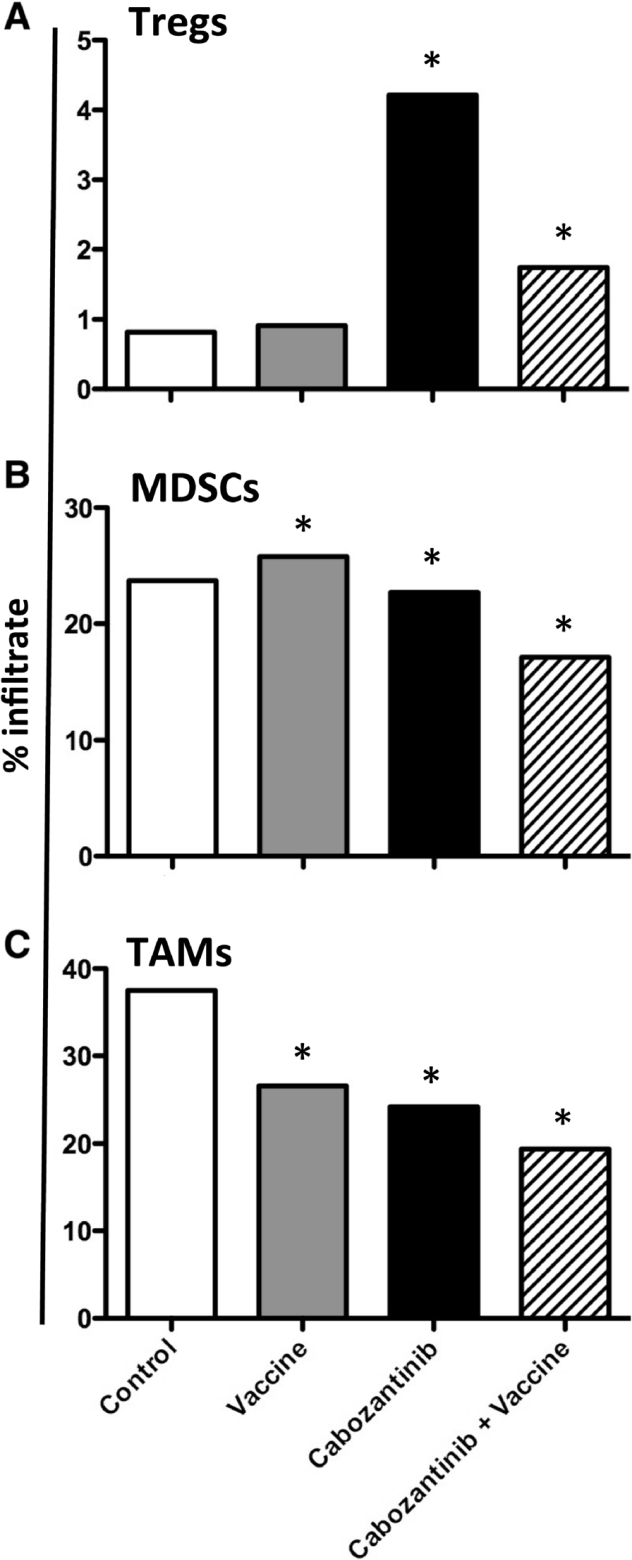
**Cabozantinib alters the tumor infiltration of negative regulatory cell subsets.** A portion of the tumors depicted in Figure 4 were homogenized into a single-cell suspension and analyzed by flow cytometry for the presence of **(A)** Tregs (CD3^+^CD4^+^CD25^+^FoxP3^+^), **(B)** MDSCs (CD11b^+^Gr-1^+^), and **(C)** TAMs (CD11b^+^Gr-1^−^). Statistical analyses were done by Kolmogorov-Smirnov test. * = *P* <0.01 upregulation relative to control. ** = *P* <0.01 downregulation relative to control.

It has been reported that cabozantinib reduces tumor vascularity, primarily through the inhibition of VEGF receptors [1,39,40]. We sought to confirm this observation in the MC38-CEA model and determine whether cabozantinib could also affect infiltration of immune cells into the tumor microenvironment when combined with a cancer vaccine. We implanted CEA-Tg C57/BL6 mice with MC38-CEA cells and treated them as indicated in Figure 4A. Immunohistochemical analysis indicated that cabozantinib treatment significantly reduced the vascular density of MC38-CEA tumors compared to tumors from control-treated mice (*P* =0.0166), and this reduction was maintained when cabozantinib was combined with MVA/rF-CEA/TRICOM (*P* =0.0249) (Figure 4B and C). In addition, tumors from mice treated with the combination of cabozantinib and MVA/rF-CEA/TRICOM showed significantly increased infiltration of CD3^+^ lymphocytes (*P* =0.0094) (Figure 4B and D, insert). Tumors from these mice did not show a significant increase in CD4^+^ T-cell infiltration (Figure 4B and E), but did show a significant increase in the number of infiltrating CD8^+^ T cells (*P* =0.0310) (Figure 4B and F). Flow cytometric analysis revealed that the tumors from mice receiving cabozantinib either alone or in combination with vaccine had a significant increase in Treg infiltration (*P* =0.01-0.005, *P* =0.002-0.001, respectively) (Figure 5A). However, cabozantinib and, to a further extent, the combination of cabozantinib and MVA/rF-CEA/TRICOM reduced the infiltration of MDSCs (*P* =0.01-0.005, *P* =0.005-0.002, respectively) (Figure 5B). Treatment with either cabozantinib or MVA/rF-CEA/TRICOM alone reduced the percentage of infiltrating tumor-associated macrophages (TAMs) (*P* =0.01–0.005), but again, the combination further reduced TAM infiltration (Figure 5C). Taken together, these data show that cabozantinib induces a more immune-permissive environment, both in the periphery and at the tumor site, and that this altered environment may be exploited by combining cabozantinib with a cancer vaccine.

### Combination with a cancer vaccine further improved cabozantinib’s antitumor activity

**Figure 6:**
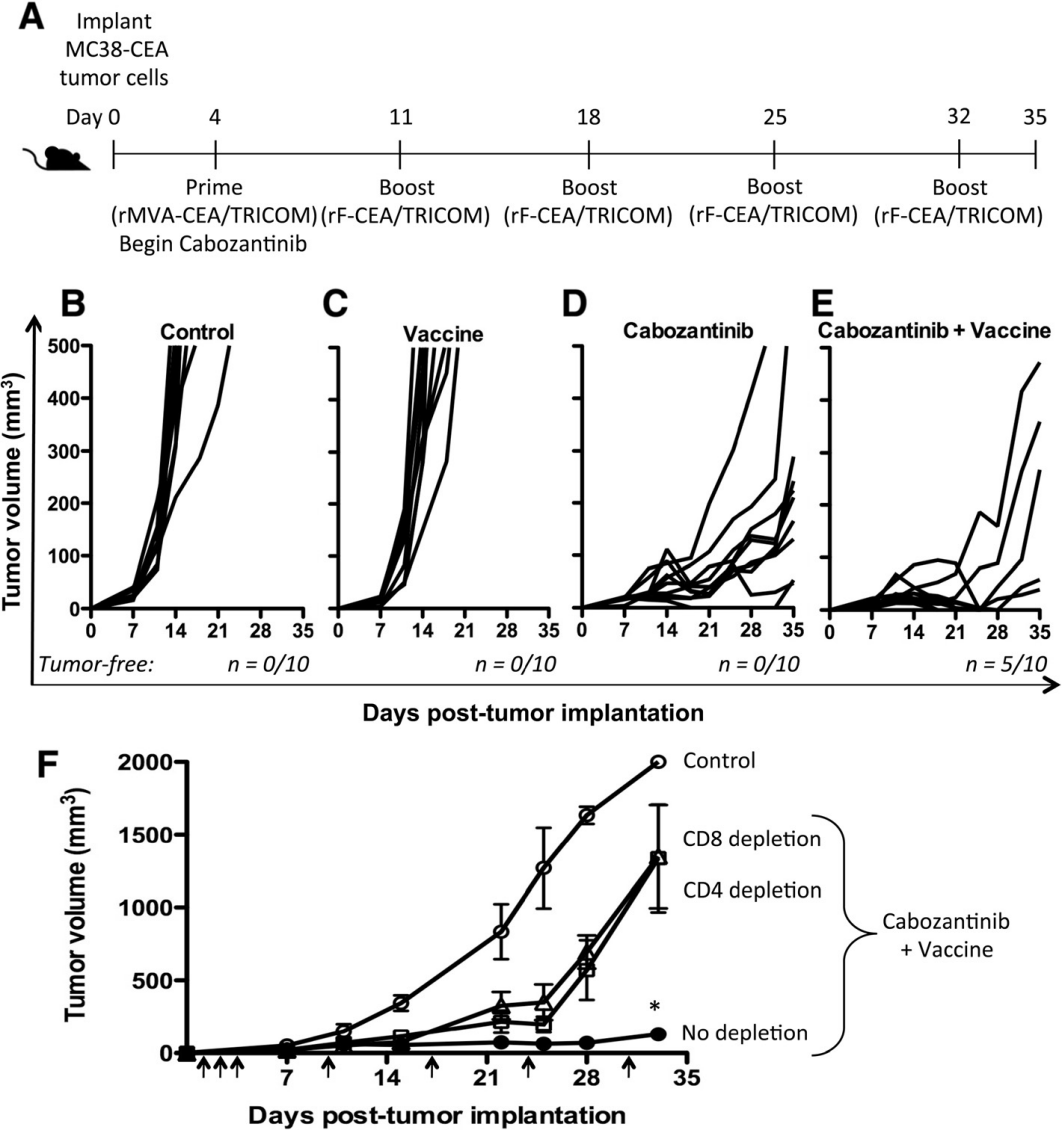
**The combination of cabozantinib and immunotherapy significantly reduced tumor growth in a T cell-dependent manner. (A)** Schema of combination therapy study (*n* =10/group). Tumor growth in mice treated with **(B)** control, **(C)** vaccine, **(D)** cabozantinib, and **(E)** combination therapy, including the number of tumor-free mice at day 35. **(F)** To determine the role of T cells in the efficacy of combination therapy, we measured tumor growth in control mice with no depletion (open circles), mice receiving combination therapy with no depletion (closed circles), and mice receiving combination therapy in the absence of CD4^+^ T cells (open squares) or CD8^+^ T cells (open triangles) (*n* =8). Depletion antibodies were administered on days 1–3, 10, 17, 24, and 31 (arrows). Tumor dimensions were measured weekly. Error bars indicate mean ± SEM. Statistical analyses were done by Student’s *t* test. * = *P* <0.0001 relative to control.

We evaluated the antitumor activity of cabozantinib in the MC38-CEA tumor model with and without the addition of MVA/rF-CEA/TRICOM (Figure 6A). Cabozantinib treatment alone significantly reduced the growth rate of MC38-CEA tumors compared to control (Figure 6B and D). In addition, cabozantinib induced tumor regression in one mouse; this tumor reappeared, however, by day 33. The combination of cabozantinib and MVA/rF-CEA/TRICOM significantly reduced the growth rate of MC38-CEA tumors compared to control and MVA/rF-CEA/TRICOM alone. Additionally, 50% of the mice treated with this combination had durable regression of their tumors and remained tumor-free through day 35 (Figure 6E). Administration of cabozantinib prior to MVA/rF-CEA/TRICOM did not further improve the antitumor efficacy of the combination (data not shown). Also, discontinuing cabozantinib after 10 days of treatment, or administering a lower dose (3 mg/kg bw/day), reduced its antitumor activity as a single agent and eliminated its synergy with MVA/rF-CEA/TRICOM (data not shown). Depleting either CD4^+^ or CD8^+^ T cells also abrogated the antitumor activity observed when cabozantinib was combined with MVA/rF-CEA/TRICOM suggesting a role for both T cell subsets in the increased efficacy of the combination treatment (Figure 6F).

## Discussion

Multiple studies have indicated that standard cancer treatments such as chemotherapy and radiation therapy can alter the phenotype of cancer cells, making them more amenable to T cell-mediated lysis (immunogenic modulation), or alter the immune landscape peripherally or in the tumor microenvironment (immune subset conditioning), indicating the potential for synergy with cancer immunotherapies [27]. We have previously demonstrated synergy between sunitinib, a small molecule inhibitor, and the MVA/rF-CEA/TRICOM cancer vaccine platform [34]. In our studies, sunitinib reduced the number and function of peripheral Tregs and MDSCs, induced lymphocyte proliferation, and increased the percentage of circulating and tumor-infiltrating CEA-specific CD8^+^ T cells. In tumor-bearing mice, the combination of sunitinib and MVA/rF-CEA/TRICOM reduced tumor growth and improved overall survival [34]. Here we sought to further these findings by examining cabozantinib, a novel RTK inhibitor, to determine if it could also induce immune subset conditioning due to an overlap of receptor inhibition with sunitinib. In addition, we hypothesized that, due to its slightly different range of RTK inhibition, cabozantinib could potentially induce immunogenic modulation, leading to even greater synergy with cancer immunotherapy.

We found that treatment with cabozantinib alone induced immune subset conditioning in the periphery. Cabozantinib treatment significantly increased the frequency of both CD4^+^ and CD8^+^ T cells in the spleen after 10 days of treatment with the increased frequency of CD8^+^ T cells being maintained through day 35 (Figure 2D). MET signaling has been shown to suppress the inflammatory response of macrophages as well as the activation and function of dendritic cells [41–43]. Cabozantinib’s ability to abrogate this suppression may influence antigen presentation by professional APCs, and therefore T-cell activation, proliferation, and trafficking. Cabozantinib not only increased the frequency of effector cells, but also significantly reduced the frequency of negative immune regulatory cells (Tregs and MDSCs) (Figure 2E and F). A recent study has reported a similar reduction in circulating Tregs in metastatic urothelial carcinoma patients treated with cabozantinib, supporting the clinical relevance of this finding [44]. It has been suggested that altering the ratio between effector and regulatory cells could create an immunostimulatory environment capable of breaking tolerance, thus allowing for the develpment of an antitumor immune response against a self-antigen [45–47]. When combined with MVA/rF-CEA/TRICOM, a cancer vaccine directed toward a self-antigen, cabozantinib increased the infiltration of lymphocytes, specifically CD8^+^ T cells, into the tumor microenvironment (Figure 4D and F). Cabozantinib likely facilitated this increased T cell infiltration through the direct reduction/normalization of the tumor vasculature (Figure 4C) as has been described by Huang, et al. [48]. In contrast, cabozantinib reduced the infiltration of negative immune regulatory cells, MDSCs and TAMs. This effect was magnified when cabozantinib was combined with MVA/rF-CEA/TRICOM, again generating a more permissive immune environment (Figure 5B and C).

Cabozantinib alone had a significant impact on the growth of MC38-CEA tumors *in vivo* (Figure 6D). This effect could be primarily attributed to cabozantinib’s significant impact on tumor vasculature (Figure 4C). Cabozantinib alone, however, did not induce durable tumor regression, without regrowth prior to day 35 of treatment. Complete tumor regression, without regrowth, was seen only when cabozantinib was combined with MVA/rF-CEA/TRICOM (Figure 6E) and required the presence of both CD4^+^ and CD8^+^ T cells (Figure 6F). This observation has been previously noted with this vaccine platform and is likely due to a need for CD4^+^ cytokine support by antigen specific CD8^+^ T cells [49]. Cabozantinib also demonstrated increased efficacy when administered prior to vaccine (data not shown). It is possible that the reduced vascular density and increased lymphocytic infiltration of the tumor, induced by cabozantinib treatment alone, was sufficient to reduce the growth rate of tumor cells but not sufficient to induce enough tumor-cell death to eliminate the tumor. The addition of a cancer vaccine further reduced the function of negative immune regulatory cells and improved the function of antigen-specific effector cells, leading to tumor eradication (Figures 3B,C,D and 6E). A lower dose of cabozantinib (3 mg/kg bw/day) did not synergize with MVA/rF-CEA/TRICOM to induce tumor regression (data not shown), suggesting that a minimum serum concentration of cabozantinib is required to achieve synergistic results. Also, if cabozantinib was discontinued after 10 days, tumor regression was not observed (data not shown), suggesting that cabozantinib levels must be maintained during vaccine treatment to achieve synergistic results.

Remarkably, despite its significant antitumor activity, cabozantinib treatment induced an increase in Treg infiltration into the tumor microenvironment, an increase that was maintained, though to a lesser degree, when cabozantinib was combined with vaccine (Figure 5A). The functional capacity of tumor infiltrating Tregs was not assayed, however, we were able to demonstrate that cabozantinib treatment significantly reduced the ability of peripheral Tregs to perform their regulatory function (Figure 3B). MVA/rF-CEA/TRICOM treatment alone similarly reduced the suppressive activity of Tregs. To our knowledge this is the first time a cancer vaccine, when used alone, has been shown to interfere with the negative regulatory capacity of these cells. The combination of cabozantinib and MVA/rF-CEA/TRICOM, however, reduced the regulatory capacity of peripheral Tregs in this mouse model to such an extent that they had no effect on the proliferation of naïve CD4^+^ T cells (Figure 3B). This suggests that while the number of Tregs in the tumor microenvironment may increase with this therapeutic regimen, their regulatory capacity may be impaired.

In these studies, we demonstrated that cabozantinib treatment could not only modulate the immune landscape both peripherally and intratumorally, but we also determined that cabozantinib could induce phenotypic changes in MC38-CEA tumor cells that increased their sensitivity to T cell-mediated lysis, hallmarks of immunogenic modulation. Specifically, cabozantinib treatment upregulated the expression of MHC-I molecules, ICAM-1, Fas, and calreticulin on tumor cells (Figure 1B). We have previously shown that radiation and chemotherapy can increase the sensitivity of tumor cells to T cell-mediated lysis through upregulation of these same molecules [31–33,37]. Indeed, cabozantinib treatment increased the sensitivity of MC38-CEA cells to lysis mediated by T cells specific for either CEA or gp70 (Figure 1C and D). The observation that cabozantinib treatment did not make MC38-CEA cells more sensitive to the cytotoxic effects of radiation (Additional file 1) suggests that cabozantinib’s alteration of the tumor cells is likely purely immunogenic. To our knowledge, this is the first time that a small molecule inhibitor has been shown to induce immunogenic modulation, and the first time that a single agent has been shown to induce both immunogenic modulation and immune subset conditioning.

## Conclusions

Combination therapy for the treatment of cancer is becoming more common as studies continue to demonstrate the complexity of cancer progression and the ability of cancer cells to become resistant to single-agent therapy, particularly that of small molecule inhibitors. Optimally, combined therapies should synergize to produce greater anticancer efficacy than that achieved by either therapy alone. The data presented here demonstrate that, in addition to its inherent antitumor properties, cabozantinib is capable of both immunogenic modulation and immune subset conditioning, two qualities displayed by therapies that synergize with cancer immunotherapy. Collectively, these data support the clinical investigation of combination therapy with cabozantinib and cancer vaccines for the treatment of solid tumors.

## Methods and materials

### Animals

Eight- to 12-week-old female C57/BL6 mice were obtained from the National Cancer Institute’s Frederick Cancer Research Facility, Frederick, MD. CEA-transgenic (CEA-Tg) mice homozygous for the expression of human CEA were generously provided by Dr. John Shively (Beckman Research Institute, City of Hope National Medical Center, Duarte, CA) and were bred and maintained at the National Institutes of Health (Bethesda, MD) [50].

### Tumor cells

Murine colon carcinoma MC38 cells expressing human CEA (MC38-CEA) were generated by retroviral transduction of MC38 cells with CEA cDNA, as previously described [51]. MC38-CEA cells were cultured in complete medium (DMEM supplemented with 10% fetal bovine serum, 2 mM glutamine, 1 mM HEPES buffer, 50 μg/mL gentamicin, 100 IU/mL penicillin, 100 μg/mL streptomycin, and 300 μg/mL G418) at 37°C/5% CO_2_.

### Drug preparation

For *in vitro* studies, cabozantinib malate salt (Exelixis Inc., South San Francisco, CA) was dissolved in DMSO (vehicle) at 1.0 mg/mL and stored at −20°C. A dose of 2.5 μg/mL was used for all *in vitro* experiments. For *in vivo* studies, cabozantinib was admixed into standard rodent diet (Research Diets, New Brunswick, NJ) at a concentration of 66.7 mg/kg of diet in order to deliver 10 mg/kg bw/day to the animals. To determine the steady-state serum level of cabozantinib achieved by delivering the drug via rodent diet, CEA-Tg C57BL/6 mice (*n* =5) were fed control diet or cabozantinib-containing diet for 10 days. Peripheral blood, acquired by retro-orbital bleeding, was analyzed for cabozantinib concentration by LC-MS.

### Poxviral vaccine constructs

Modified vaccinia Ankara (MVA) and recombinant fowlpox (rF) viruses containing transgenes for the murine costimulatory molecules B7-1, ICAM-1, and LFA-3 (designated TRICOM) in combination with the human CEA transgene (MVA/rF-CEA/TRICOM) have been previously described [52]. For *in vivo* studies, a priming dose of MVA-CEA/TRICOM was administered s.c., with weekly boosts of rF-CEA/TRICOM, both at 1 × 10^8^ plaque-forming units/mouse/dose.

### Western blotting

MET and VEGFR2 expression was determined by western blot using rabbit polyclonal antibodies to MET and VEGFR2 (Abcam, Cambridge, MA). MC38-CEA cells were lysed using Cell Lysis Buffer containing 1 mM PMSF (Cell Signalling, Danvers, MA) and 10 μL/mL HALT Protease/Phosphatase Inhibitor Cocktail (Thermo Scientific, Rockford, IL) according to the manufacturer’s protocol, blots were imaged using the Odyssey Infrared Imaging System (LI-COR Biosciences, Lincoln, NE).

### *In vitro* proliferation analysis

To investigate the effect of cabozantinib on MC38-CEA cell proliferation, cells were plated in 24-well plates and treated with cabozantinib or vehicle (DMSO) *in vitro* for 1, 3, or 5 days, then harvested and counted using trypan blue counterstaining. To examine the effects of radiation on cabozantinib-treated MC38-CEA cells, cells were treated with cabozantinib for 24 h, then irradiated (0 or 5 Gy) by exposure to a Cs-137 source using a Gammacell-1000 (AECL/Nordion, Kanata, Ontario, Canada), and then replated. Cells were again harvested at 24, 48, and 72 h post-irradiation, counted, and assayed for viability using trypan blue.

### *In vitro* phenotypic analysis

To analyze the effect of cabozantinib on *in vitro* expression of immune-relevant proteins, MC38-CEA cells were treated with cabozantinib or vehicle for 24 h, then stained with the following antibodies: CD66e/CEA-FITC (AbD Serotec, Raleigh, NC), H-2K^b^-APC (eBioscience, San Diego, CA), H-2D^b^-PE, CD54/ICAM-I-PE, CD95/Fas-FITC (BD Biosciences, San Jose, CA) and calreticulin-APC (R&D Systems, Minneapolis, MN). 7AAD (BD Biosciences) staining was used to determine cell viability. Cells were incubated with the antibodies for 30 min at 4°C, acquired on an LSR II flow cytometer (Becton Dickinson, Franklin Lakes, NJ), and analyzed using FlowJo software (TreeStar, Inc., Ashland, OR).

### Cytotoxic T lymphocyte killing assay

To evaluate cabozantinib’s ability to alter the sensitivity of MC38-CEA cells to lysis by CTLs, cells were treated with cabozantinib, vehicle or left untreated for 24 h, after which they were harvested and used as targets in a standard CTL assay. Cells were labeled with ^111^In-labeled oxyquinoline (Medi-Physics Inc., Arlington Heights, IL) and coincubated in 96-well round-bottom plates at 37°C/5% CO_2_ with T572- or gp70-specific effector cells in the absence of cabozantinib at an effector:target ratio of 30:1. The H-2D^b^-restricted CEA-specific CD8^+^ CTL line, T572, recognizes the peptide epitope CEA_572–579_, as previously described [53]. The H-2K^b^-restricted gp70-specific CD8^+^ CTL line, gp70, recognizes the peptide epitope p15e_604–611_ of glycoprotein 70 of an endogenous murine retrovirus, as previously described [54]. After 18 h, supernatants were harvested and analyzed for the presence of ^111^In using a WIZARD2 Automatic Gamma Counter (PerkinElmer, Waltham, MA). The percentage of tumor lysis was calculated as follows: % tumor lysis = [(experimental cpm – spontaneous cpm)/(maximum cpm – spontaneous cpm)] × 100.

### *In vivo* studies

#### Analysis of immune cell populations

To analyze immune cell populations in the presence or absence of cabozantinib, CEA-Tg C57BL/6 mice (*n* =5/group) were fed control or cabozantinib-containing diet for 10 or 35 days. Spleens were harvested and adjusted to a single-cell suspension. Red blood cells were removed using ACK Lysing Buffer (Quality Biologicals Inc., Gaithersburg, MD). Remaining splenocytes were blocked with mouse Fc Block (BD Biosciences) for 30 min at 4°C, then stained with the following antibodies: CD3e-V500, CD4-AF700, CD8a-Pacific Blue, CD25-FITC, CD11b-V500, Gr-1-AF700, CD49b-FITC, CD19-PE-Cy7, CD11c-PerCP-Cy5.5 (BD Biosciences) and MHC II-APC (eBioscience) for 60 min at 4°C. For intracellular staining of cells with FoxP3-PE, cells were incubated with Fixation/Permeabilization solution for 16 h at 4°C, then incubated with the antibody in Permeabilization Buffer (eBioscience) for 60 min at room temperature. All samples were acquired on an LSR II flow cytometer and analyzed using FlowJo software.

### Immune cell function assays

To assess the effect of cabozantinib and MVA/rF-CEA/TRICOM on immune cell function, CEA-Tg C57BL/6 mice were divided into 4 groups (*n* =5/group): (a) control, (b) cabozantinib alone, (c) MVA/rF-CEA/TRICOM alone, and (d) cabozantinib + MVA/rF-CEA/TRICOM. Mice receiving cabozantinib were fed cabozantinib-containing diet on days 0–35. Mice receiving MVA/rF-CEA/TRICOM received a priming vaccination with MVA-CEA/TRICOM on day 0, then booster vaccinations with rF-CEA/TRICOM on days 7 and 14. Spleens were harvested on day 35. To examine the function of Tregs, splenocytes were purified by Histopaque gradient (Sigma-Aldrich, St. Louis, MO), then CD4^+^CD25^+^ Tregs were purified using a Regulatory T Cell Isolation Kit (Miltenyi Biotec, Auburn, CA) according to the manufacturer’s instructions. Purified Tregs were plated with antigen presenting cells (APCs), irradiated (30 Gy) splenocytes from naïve C57BL/6 mice, CD4^+^ T cells, purified from naïve C57BL/6 mice using a CD4+ T Cell Isolation Kit (Miltenyi Biotec), and anti-CD3 cross-linking antibody (BD Biosciences) for 72 h. [^3^H]-thymidine was added to the culture for the last 12 h of incubation. Control wells containing Tregs, APCs, and anti-CD3 without CD4^+^ cells were used to determine the background level of Treg proliferation. Control wells containing CD4^+^ cells and Concanavalin A (Sigma-Aldrich) were used to determine the maximum level of CD4^+^ cell proliferation. Cells were harvested using a Tomtec cell harvester (Wallac Inc., Gaithersburg, MD) and [^3^H]-thymidine incorporation was measured using a Wallac 1205 Betaplate MicroBeta Counter (Wallac Inc.) To examine cytokine production by effector T cells, splenocytes were incubated with CEA_572–579_ peptide (GIQNSVSA) (CPC Scientific, Sunnyvale, CA) for 7 days then purified on a Histopaque gradient. Effector cells were plated with APCs in the presence of CEA_572_ peptide for 24 h. Supernatants were collected and analyzed for the presence of cytokines using a mouse Th1/Th2 Cytometric Bead Array (BD Biosciences). Data were acquired using a FACScan flow cytometer and analyzed using BD CBA analysis software (Becton Dickinson).

### Characterization of the tumor microenvironment

To investigate the effect of cabozantinib and MVA/rF-CEA/TRICOM on tumor vascularity and immune cell infiltration, CEA-Tg C57BL/6 mice were implanted with 3 × 10^5^ MC38-CEA cells s.c. in the right flank. Fourteen days post tumor implantation, when tumors were established (~300 mm^3^), mice were divided into 4 groups (*n* =2/group): (a) control, (b) cabozantinib alone, (c) MVA/rF-CEA/TRICOM alone, and (d) cabozantinib + MVA/rF-CEA/TRICOM. Mice treated with cabozantinib were fed cabozantinib-containing diet on days 14–28. Mice treated with MVA/rF-CEA/TRICOM received a priming vaccination with MVA-CEA/TRICOM on day 14 and a booster vaccination with rF-CEA/TRICOM on day 21. On day 28, tumors were harvested with a portion of each tumor being fixed with Z-Fix (Anatech Ltd., Battle Creek, MI), frozen in OTC (Electon Microscopy Sciences, Hatfield, PA), or dissociated according to the protocol for preparation of single-cell suspensions from implanted mouse tumors (Miltenyi Biotec). Fixed tumor sections were stained with antibodies to von Willebrand factor at 1:1200 and CD3 at 1:1000 (Dako, Carpinteria, CA). Frozen tumor sections were stained with rat monoclonal antibodies to CD4 and CD8 at 1:1500 and 1:700, respectively (Novus Biologicals, Littleton, CO). Control sections were stained with matched isotype antibodies. Entire tumor sections were digitally scanned by an Aperio ScanScope CS scanning system and analyzed by Aperio ImageScope Viewer software (Aperio Technologies Inc., Vista, CA) excluding necrotic regions. Positive tumor regions were determined using the Microvessel Analysis v1 and Positive Pixel Count v9 algorithms, respectively and are depicted as number of vessels or cells/tumor. Dissociated tumor was stained for flow cytometric analysis using the same antibodies and protocol as the splenic immune cell population analysis with the inclusion of CD45-BV605 (ebioscience).

### Antitumor studies

To examine the antitumor effects of cabozantinib and MVA/rF-CEA/TRICOM, CEA-Tg C57BL/6 mice were implanted with 3 x 10^5^ MC38-CEA cells s.c. in the right flank on day 0. On day 4, when tumors became palpable, mice were divided into 4 groups (*n* =10/group): (a) control, (b) cabozantinib alone, (c) MVA/rF-CEA/TRICOM alone, and (d) cabozantinib + MVA/rF-CEA/TRICOM. Mice treated with cabozantinib were fed cabozantinib-containing diet on days 4–35. Mice treated with MVA/rF-CEA/TRICOM received a priming vaccination with MVA-CEA/TRICOM on day 4, then weekly booster vaccinations with rF-CEA/TRICOM. Tumor dimensions were measured twice weekly and tumor volumes were calculated as follows: (length × width^2^)/2. For depletion studies, MC38-CEA-bearing CEA-Tg C57BL/6 mice (*n* =8/goup) were given either anti-CD4 (GK 1.5) or anti-CD8 (Lyt 2.2) hybridomas i.p. on days 1–3, then at weekly intervals, or left untreated. Depletion was confirmed by flow cytometric analysis of peripheral blood. All mice, except for those in the control group, received cabozantinib-containing diet on days 4–33, a priming vaccination with MVA-CEA/TRICOM on day 4, then weekly booster vaccinations with rF-CEA/TRICOM.

### Statistical analysis

GraphPad Prism 5 statistical software (GraphPad Software, La Jolla, CA) was used to measure 2-tailed unpaired Student’s *t* tests for differences between groups, with a 95% confidence interval. All data represent the mean ± SEM for the indicated number of replicates or individual mice. FlowJo software was used to determine significant differences in the distribution of flow cytometry data using the Kolmogorov-Smirnov test.

## Additional file

Additional file 1:
**Cabozantinib treatment does not increase the sensitivity of MC38-CEA cells to external-beam radiation.**

## Abbreviations

RTKs: Receptor tyrosine kinases
FDA: Food and Drug Administration
CTLA-4: Cytotoxic T-lymphocyte-associated protein 4
CEA: Carcinoembryonic antigen
MHC: Major histocompatibility complex
ICAM-1: Intercellular Adhesion Molecule 1
CTLs: Cytotoxic T lymphocytes
CEA-Tg: C57/BL6 mice transgenic for human CEA
Tregs: T regulatory cells
MDSCs: Myeloid derived suppressor cells
MVA: Modified vaccinia Ankara
rF: Recombinant fowlpox virus
TRICOM: Triad of costimulatory molecules including B7-1, ICAM-1, LFA-3
LFA-3: Lymphocyte function-associated antigen 3
MVA-CEA/TRICOM: MVA expressing human CEA and TRICOM
rF-CEA/TRICOM: rF expressing human CEA and TRICOM
MVA/rF-CEA/TRICOM: Prime/boost vaccination strategy administering MVA-CEA/TRICOM as a priming vaccination and rF-CEA/TRICOM as the booster vaccinations
IFN-γ: Interferon γ
TNF-α: Tumor necrosis factor α
TAMs: Tumor associated macrophages
DMSO: Dimethylsufoxide
LC-MS: Liquid chromatography–mass spectrometry
PMSF: Phenylmethanesulfonyl fluoride
FITC: Fluorescein isothiocyanate
PE: Phycoerythrin
APC: Allophycocyanin
cpm: Counts per minute
APCs: Antigen presenting cells
SEM: Standard error of the mean
