# Supplementary figures and images for: Dual effects of a targeted small-molecule inhibitor (cabozantinib) on immune-mediated killing of tumor cells and immune tumor microenvironment permissiveness when combined with a cancer vaccine
Source: J Transl Med. 2014 Nov 13;12:294. doi: 10.1186/s12967-014-0294-y (PMC4236498; doi:10.1186/s12967-014-0294-y)

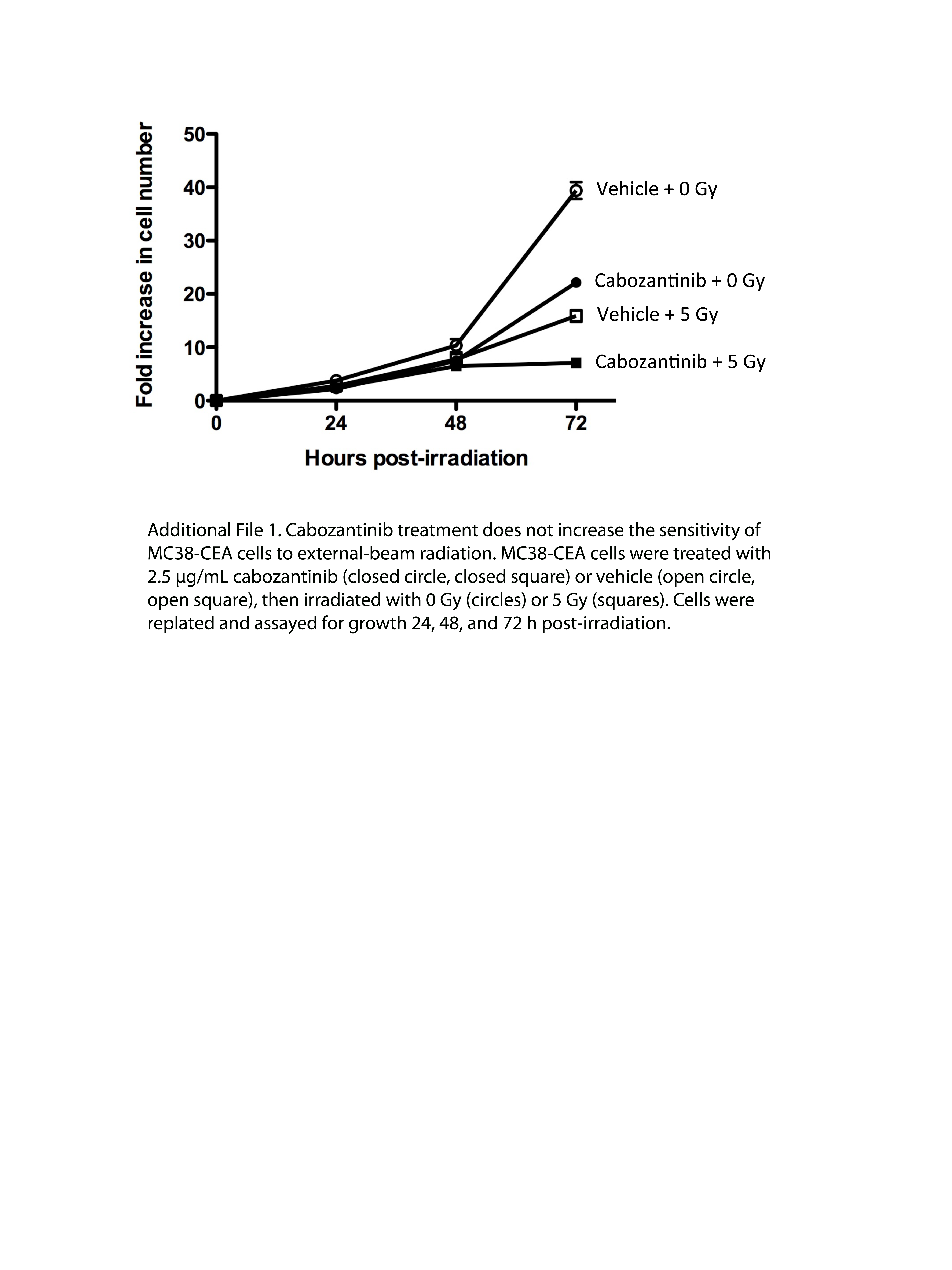

Supplement: Additional file 1: — Cabozantinib treatment does not increase the sensitivity of MC38-CEA cells to external-beam radiation. [file 12967_2014_294_MOESM1_ESM.tif]
